# Chemical, Thermal and Antioxidant Properties of Lignins Solubilized during Soda/AQ Pulping of Orange and Olive Tree Pruning Residues

**DOI:** 10.3390/molecules26133819

**Published:** 2021-06-23

**Authors:** María E. Eugenio, Raquel Martín-Sampedro, José I. Santos, Bernd Wicklein, David Ibarra

**Affiliations:** 1 Forest Research Center (INIA, CSIC), Ctra. de la Coruña Km 7.5, 28040 Madrid, Spain; mariaeugenia@inia.es (M.E.E.); raquel.martin@inia.es (R.M.-S.); 2 NMR of Facility of Research (SGIker), University of the Basque Country (UPV/EHU), Avenida Tolosa 72, 20018 Donostia-San Sebastián, Spain; joseignacio.santosg@ehu.eus; 3 Materials Science Institute of Madrid (ICMM), Consejo Superior de Investigaciones Científicas (CSIC), Sor Juana Inés de la Cruz 3, 28049 Madrid, Spain; bernd@icmm.csic.es

**Keywords:** lignin purity and composition, lignin structural characterization, lignin thermal properties, lignin antioxidant properties, olive tree pruning, orange tree pruning, soda/AQ pulping

## Abstract

Some agroforestry residues such as orange and olive tree pruning have been extensively evaluated for their valorization due to its high carbohydrates content. However, lignin-enriched residues generated during carbohydrates valorization are normally incinerated to produce energy. In order to find alternative high added-value applications for these lignins, a depth characterization of them is required. In this study, lignins isolated from the black liquors produced during soda/anthraquinone (soda/AQ) pulping of orange and olive tree pruning residues were analyzed by analytical standard methods and Fourier-transform infrared spectroscopy (FTIR), nuclear magnetic resonance (solid state ^13^C NMR and 2D NMR) and size exclusion chromatography (SEC). Thermal analysis (thermogravimetric analysis (TGA), differential scanning calorimetry (DSC)) and antioxidant capacity (Trolox equivalent antioxidant capacity) were also evaluated. Both lignins showed a high OH phenolic content as consequence of a wide breakdown of β-aryl ether linkages. This extensive degradation yielded lignins with low molecular weights and polydispersity values. Moreover, both lignins exhibited an enrichment of syringyl units together with different native as well as soda/AQ lignin derived units. Based on these chemical properties, orange and olive lignins showed relatively high thermal stability and good antioxidant activities. These results make them potential additives to enhance the thermo-oxidation stability of synthetic polymers.

## 1. Introduction

Agroforestry residues are one of the major resources of unexploited potential lignocellulosic feedstocks. Most of these residues are underutilized or burned in situ, generating serious environmental pollutions. Thus, the circular bioeconomy, as a way to reach a sustainable development, should be a chance to manage these lignocellulosic residues towards the production of energy and high added-value products. In addition, the success of this target should have a significant impact on the mitigation of both petroleum consumption and greenhouse gas (GHG) emissions.

An interesting and abundant agroforestry residue is orange tree pruning, the result of trimming away unneeded branches of orange trees. Orange tree cultivation is significant in Mediterranean countries, including Spain, where 2.82 × 10^6^ tons of oranges are produced annually [1]. This agricultural activity generates a quantity of orange tree pruning residue around 2.25 × 10^6^ tons each year [1]. In the same way, Spain is also the main olive oil producer worldwide, with about 2.3 M ha of olive trees cultivated that generates 1.3 tons ha^−1^ year^−1^ (3.0 tons ha^−1^ biennial pruning) of olive tree pruning residue [2]. These tree pruning residues, which include a main woody fraction and a remaining fraction consisting of leaves and thin branches, have been extensively evaluated for their valorization due to its high carbohydrates (cellulose and hemicellulose) content. Then, cellulose has been used for production of bioethanol [3], cellulosic pulp and advanced materials such as nanocellulose, among others [1,4,5,6]. Hemicelluloses have been exploited for production of xylitol and xylooligosacharides [7,8]. However, lignin-enriched residues generated during these processes, solubilized in black liquors from pulp and paper industry or as non-fermentable residues resulting from bioethanol production, are currently underutilized, being normally incinerated to generate energy i.e., heat and electricity, which supplies part of the demands of the pulp and paper and bioethanol industries.

Lignin is a complex aromatic macromolecule composed by p-hydroxyphenyl (H) (from p-coumaryl alcohol), guaiacyl (G) (from coniferyl alcohol), and syringyl (S) (from sinapyl alcohol) phenylpropane units [9]. These units are linked through a variety of inter-units linkages, including aryl ether and carbon–carbon (C–C) bonds [9]. Among them, β-aryl ether linkage (β-*O*-4’) represents the most abundant bond, followed by resinol (β-β’) and phenylcoumaran (β-5’) among others. Depending on the lignocellulosic feedstock, extraction process and conditions, lignin displays a range of properties, i.e., aromatic structure, chemical functionalities, hydrophobicity, thermal stability and treatability, antioxidant, etc., which makes it interesting as a feedstock to produce chemicals and materials [10]. Then, lignin has been assessed for polymer and material applications such as carbon materials, resins, hydrogels, and polymer modifiers and/or as a source to produce bulk and fine chemicals such as benzene, toluene and xylene (BTX), phenols and vanillin [10,11]. Therefore, in addition to helping to the competitive and sustainable production of energy and high added-value products from carbohydrates, the valorization of lignin-enriched residues generated during carbohydrates transformation will also contribute for the implementation of the circular bioeconomy, which aims to maximize the usage and value of all raw materials, products, and residues.

Kraft pulping with NaOH and Na_2_S is the alkaline process most widely employed for delignification of woods, i.e., hardwoods and softwoods, in the pulp and paper production [12]. Yearly, 120,000 tons of kraft lignin is generated [13]. On the other hand, soda/anthraquinone (soda/AQ) pulping, with AQ as a pulping additive to limit the carbohydrate degradation [12], is generally used for agriculture residues [14]. In this sense, soda/AQ pulping has been extensively used to produce paper pulp from orange and olive tree pruning residues [1,6]. Although smaller volumes of soda lignin are produced annually (5000 tons) compared to kraft lignin [13], soda lignin is sulfur-free, which makes it more attractive for the production of bio-based products. Nevertheless, prior to its valorization, a thorough knowledge of the purity, the type and proportion of the different inter-unit linkages (either native or derived extraction process linkages), the content of syringyl and guaiacyl units, the molecular weight, the hydroxyl proportion, and the thermal behavior is required to select the best way for lignin valorization. In this sense, multitude of studies have analyzed different residual lignins to elucidate its features depending on the biomass origin, i.e., hardwood, softwood, and non-woody materials [15,16,17], and the extraction technology, i.e., kraft pulping, organosolv, acid hydrolysis and steam explosion [18,19,20,21]. Lignins solubilized during soda/AQ pulping have also been characterized [22,23,24].

In this study, lignins solubilized during soda/AQ pulping of orange and olive tree pruning residues were recovered and their chemical composition and structural features were analyzed by analytical standard methods and Fourier-transform infrared spectroscopy (FTIR), nuclear magnetic resonance (solid state ^13^C NMR and 2D NMR) and size exclusion chromatography (SEC). Thermal analysis (thermogravimetric analysis (TGA), differential scanning calorimetry (DSC)) and antioxidant abilities (Trolox equivalent antioxidant capacity) were also evaluated. According to all this information, possible valorization ways are discussed for these lignins.

## 2. Results and Discussion

### 2.1. Chemical Composition of Lignins

Soda/AQ pulping was carried out using orange and olive tree pruning residues as raw materials. The resulting soda/AQ pulps were filtered, and the black liquors containing solubilized lignins recovered. Then, acidification at pH 2.5 of soda/AQ black liquors generated orange and olive soda/AQ lignins (denoted as soda/AQ-orange and soda/AQ-olive lignins, respectively). Orange lignin sample showed a lignin content of 75.1% (64.5 ± 1.6% of acid insoluble lignin and 10.6 ± 0.6% of acid soluble lignin), whereas olive lignin sample exhibited a lignin content of 69.9% (61.3 ± 0.22% of acid insoluble lignin and 8.6 ± 0.0% of acid soluble lignin). Some carbohydrates impurities were also determined in both lignins, being much higher in soda/AQ-olive lignin (11.0 ± 0.0% glucan, 4.7 ± 0.0% xylan and 1.6 ± 0.0% arabinan) compared to soda/AQ-orange lignin (2.7 ± 0.1% glucan, 3.1 ± 0.1% xylan and 0.6 ± 0.0% arabinan). Domínguez-Robles et al. [22] also described the presence of carbohydrates in soda lignins from agricultural residues such as wheat straw and barley straw as well as soda/AQ lignins from fast growing plants such as *Leucaena leucocephala* and *Chamaecytisus proliferus*. Part of the carbohydrates in the lignocellulosic materials is dissolved during alkaline processes, starting immediately when the lignocellulosic fibres come in contact with the alkaline pulping liquor and proceeding rapidly even at temperatures around 100 °C, especially the xylans. At temperatures about 170 °C, or higher, a random alkaline hydrolysis of glucosidic bonds may also take place [12]. These dissolved carbohydrates can partially precipitate during lignin precipitation due to their lower solubility under acidic conditions, explaining the carbohydrates content determined in both lignin samples [18]. Nevertheless, they can also be attributed to lignin-carbohydrate complexes [25].

### 2.2. FTIR Spectra Analysis of Lignins

**Figure 1:**
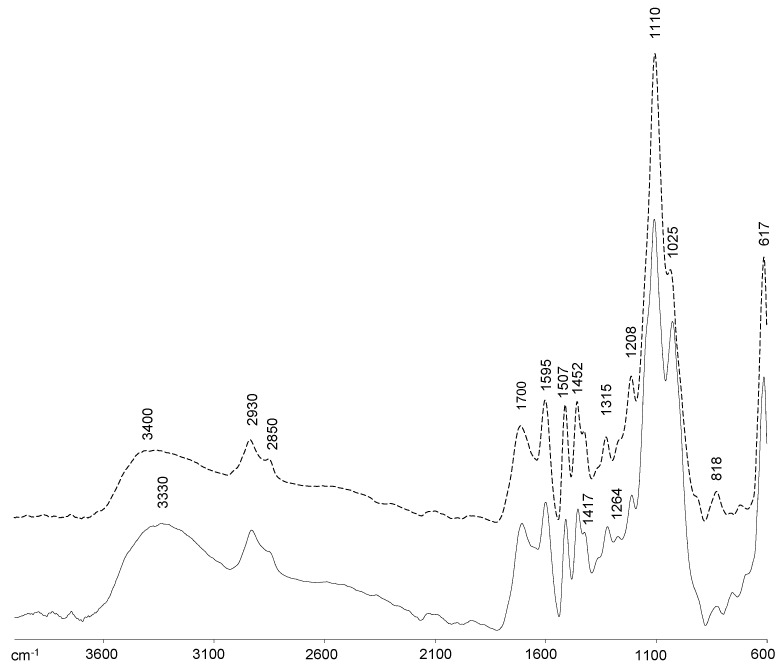
FTIR spectra, 4000–600 cm^−1^ region, of soda/AQ-orange lignin (discontinuous line) and soda/AQ-olive lignin (continuous line).

The FTIR spectra of orange and olive soda/AQ lignins are showed in Figure 1, being the visible bands identified according to previous studies [21,22,23,26,27,28]. Both spectra displayed characteristic lignin patterns, with a broad band at 3400–3300 cm^−1^ attributed to the O–H stretching vibration in aromatic and aliphatic lignin structures. The bands at 2930 cm^−1^ and 2850 cm^−1^ are associated to the symmetrical and asymmetrical C–H stretching in the methyl and methylene groups, respectively, together with the band at 1455 cm^−1^ associated to the C–H asymmetric vibrations and deformation (asymmetric in methyl and methylene). The absorption intensity at 1700 cm^−1^, corresponding to the carbonyl in the unconjugated ketones and ester groups stretching from lignin oxidation [26], was also clearly visible in both lignin samples. Nevertheless, carbonyl groups in hemicelluloses that are remaining in both lignin samples as impurities (Section 2.1) could also be contributing to this absorption [22].

Both lignin spectra displayed bands at 1595 cm^−1^, 1507 cm^−1^, and a shoulder at 1417 cm^−1^ corresponding to aromatic skeleton lignin vibrations. Other bands were associated to syringyl (S) and guaiacyl (G) units, including bands at 1315 cm^−1^ (aromatic ring breathing, S and G condensed units), 1264 cm^−1^ (G ring breathing with C=O stretching), 1208 cm^−1^ (G ring breathing with C–C, C–O, and C=O stretching), 1110 cm^−1^ (C–H bond deformation in S units), 1025 cm^−1^ (C–H bond deformation in G units) and 819 cm^−1^ (C–H out of plane deformation of S units). These lignin bands are typical of lignins from hardwoods such as eucalypt, poplar, black locust and elm materials [19,20,27,29].

In addition to carbonyl groups from hemicelluloses absorbing at 1700 cm^−1^, other bands in both lignin spectra can also reflect the carbohydrate impurities determined by chemical composition analysis (Section 2.1). Thus, cellulose and hemicellulose bands at 1110 cm^−1^ (C–OH skeletal vibration) and 1025 cm^−1^ (C–O stretching vibration) were observed. Finally, the signal at 617 cm^−1^ is attributed to C–S bending generated from the use of H_2_SO_4_ during the precipitation of lignins from soda/AQ black liquors [22].

### 2.3. Solid State ^13^C NMR Spectra Analysis of Lignins

**Figure 2:**
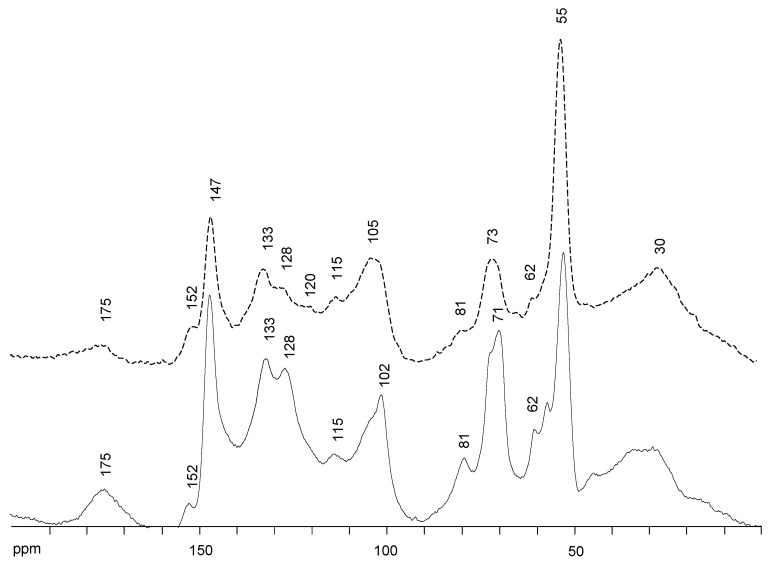
^13^C NMR spectra, δ_C_ 200.0–0.0 ppm, of soda/AQ-orange lignin (discontinuous line) and soda/AQ-olive lignin (continuous line).

The ^13^C NMR spectra of orange and olive soda/AQ lignins are displayed in Figure 2, being the signals identified based on those described in bibliography [22,26,29,30]. In accordance with FTIR patterns (Section 2.2), both ^13^C NMR spectra exhibited a signal at δ_C_ 175 ppm, especially in soda/AQ-olive lignin, assigned to carbonyl groups and aliphatic COOR from lignin oxidation during alkaline pulping [31]. Nevertheless, hemicelluloses impurities can also contribute to this signal [32]. Moreover, the aromatic regions (around δ_C_ 152–95 ppm) of both spectra were dominated by signals corresponding to phenolic units. Then, the signals at δc 147 ppm, associated to C_3_ and C_5_ in S units and C_3_ and C_5_ in G units, and at δc 133 ppm, endorsed to C_1_ and C_4_ in S units and C_1_ in G units, showed a great intensity. Contrary, only a small shoulder at δ_C_ 152 ppm from non-phenolic units was visible in both spectra. This high content of phenolic units observed by ^13^C NMR in both lignins (slightly higher for soda/AQ-orange lignin (317.6 ± 9.2 g GAE mg^−1^ lignin) than soda/AQ-olive lignin (294.3 ± 1.6 g GAE mg^−1^ lignin) after reaction with Folin–Ciocalteau reagent) indicates an abundant degradation of them produced during soda/AQ pulping. As it is well known, the β-*O*-4’ substructures is the dominant linkage in native lignin [9], representing around 50−60% of all linkages. Alkaline pulping processes, either kraft or soda pulping, generate phenolic units by cleaving β-*O*-4’ ether bonds, which help to solubilize lignin [23,24,28].

Other signals in the aromatic region of both spectra were also visible at δ_C_ 128 ppm, C_2_ and C_6_ in p-hydroxyphenyl (H) units; δ_C_ 120 ppm, C_6_ in G units; δ_C_ 115 ppm, C_5_ in G units; and δ_C_ 102 ppm, C_2_ and C_6_ in S units. Nevertheless this signal is overlapped by C_1_ in hemicellulose, or shifted to δ_C_ 105 ppm of C_1_ in cellulose [32,33], according to the carbohydrates contamination determined by chemical composition analysis (Section 2.1).

Regarding to oxygenated aliphatic region (around δ_C_ 95–50 ppm), lignin signals from native β-*O*-4’ substructure (A), including signals at δ_C_ 81 ppm for C_β_ in β-*O*-4’, δ_C_ 73 ppm for C_α_ in β-*O*-4’ and δ_C_ 62 ppm for C_γ_ in β-*O*-4’ were detected in both spectra. A signal at δ_C_ 71 ppm for C_γ_ in native β-β’ resinol substructure (B) was also visible, together with a signal at δ_C_ 56 ppm corresponding to methoxyl groups (–OCH_3_). Nevertheless, the existence of carbohydrates signals in this region complicates the interpretation of lignin substructures [32,33]. Then, in agreement with the carbohydrates impurities determined in both soda/AQ lignin samples (Section 2.1), cellulose signals at δ_C_ 81 ppm of C_4_ (amorphous), δ_C_ 73 ppm of C_2_, C_3_, C_5_ and δ_C_ 62 ppm of C_6_ (amorphous), and hemicelluloses signals at δ_C_ 73 ppm of C_2_, C_3_, C_5_ and δ_C_ 62 ppm of C_6_ can interfere with the signals identified for lignin substructures. 2D NMR analysis was applied for soda/AQ lignins analysis (Section 2.4) to resolve the lignin and carbohydrates signals overlapping.

Finally, the non-oxygenated alyphatic region (around δ_C_ 50–0 ppm) showed a broad signal at δ_C_ 30 ppm, generally attributed to alkyl carbons such as the γ-methyl, as well as the α- and β- methylene groups in n-propyl side chains of lignins, the acetyl of hemicelluloses and saturated aliphatic moieties associated with lipid extractives [32].

### 2.4. 2D NMR Spectra Analysis of Lignins

**Figure 3:**
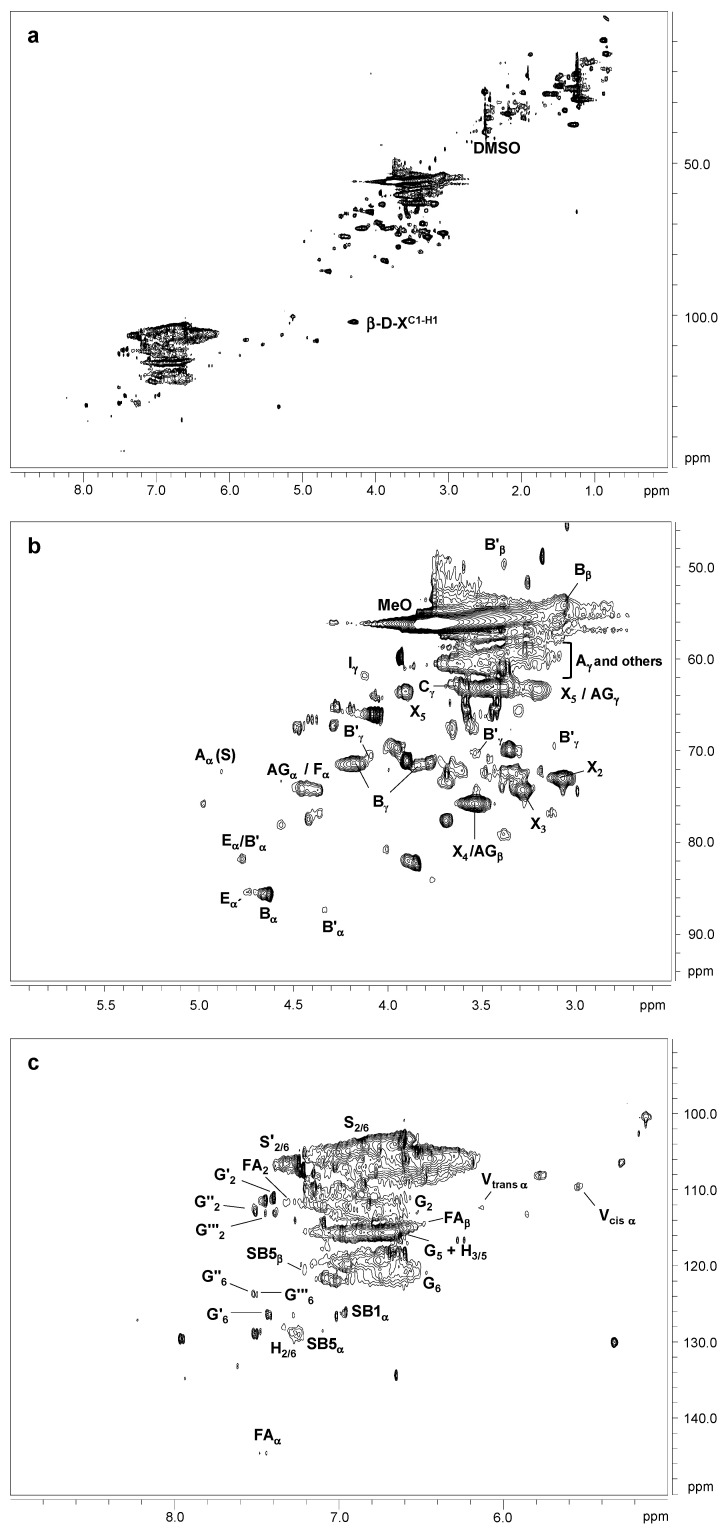
HSQC 2D-NMR spectra of soda/AQ-orange lignin. (**a**) whole spectrum, δ_C_/δ_H_ 0.0–150.0/0.0–9.0; (**b**) aliphatic oxygenated region, δ_C_/δ_H_ 45.0–95.0/2.5–6.0 ppm; (**c**) aromatic region, δ_C_/δ_H_ 90.0–150.0/5.0–9.0 ppm.

**Figure 4:**
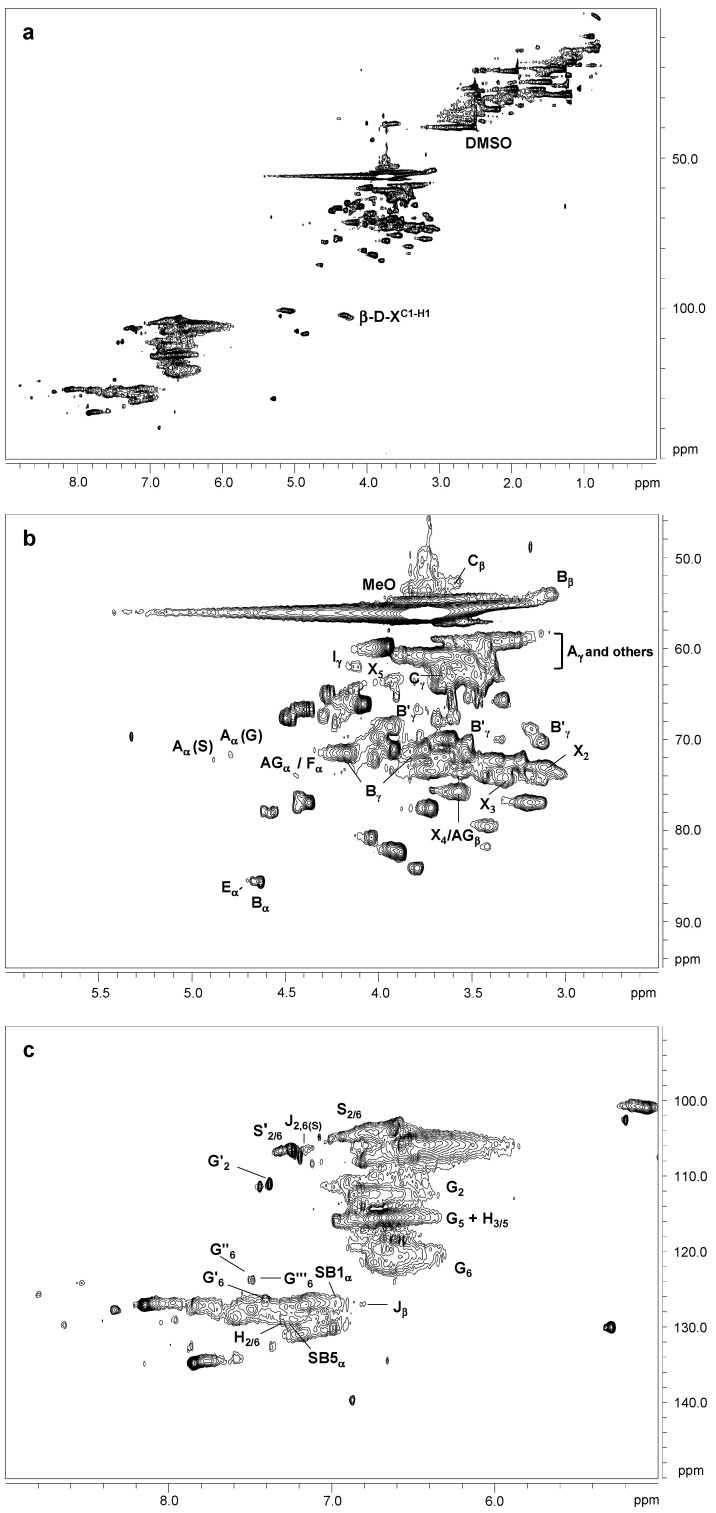
HSQC 2D-NMR spectra of soda/AQ-olive lignin. (**a**) whole spectrum, δ_C_/δ_H_ 0.0–150.0/0.0–9.0; (**b**) aliphatic oxygenated region, δ_C_/δ_H_ 45.0–95.0/2.5–6.0 ppm; (**c**) aromatic region, δ_C_/δ_H_ 90.0–150.0/5.0–9.0 ppm.

**Figure 5:**
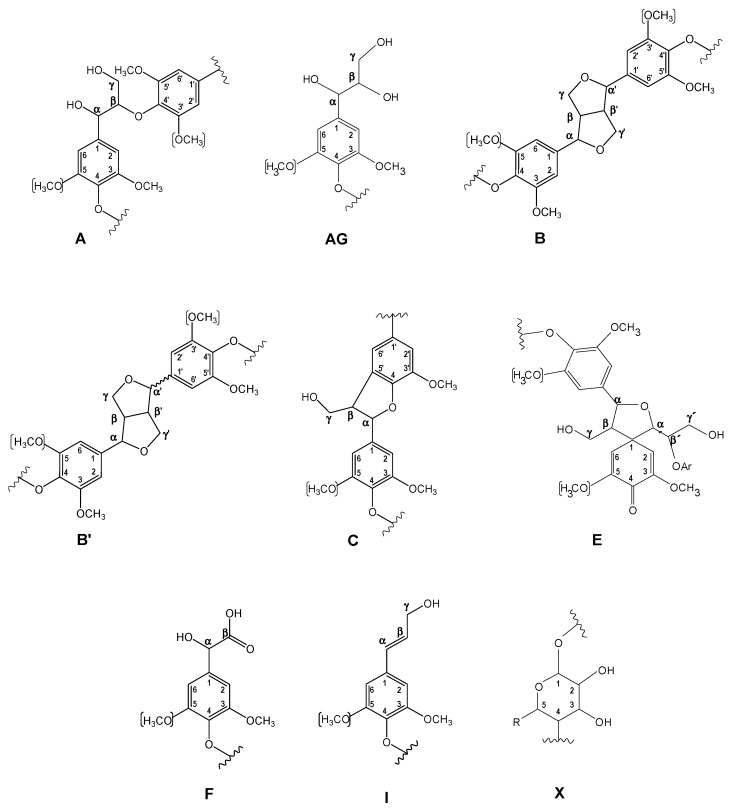
Main lignin and carbohydrate substructures identified in aliphatic oxygenated region of orange and olive soda/AQ lignins: (**A**), β-*O*-4’ alkyl-aryl ether; (**AG**), aryl-glycerol; (**B**), resinols; (**B’**), epiresinol; (**C**), phenylcoumarans; (**E**), spirodienones; (**F**), Ar–CHOH–COOH; (**I**), cinnamyl alcohol end-groups; (**X**), xylopyranose (R, OH).

**Figure 6:**
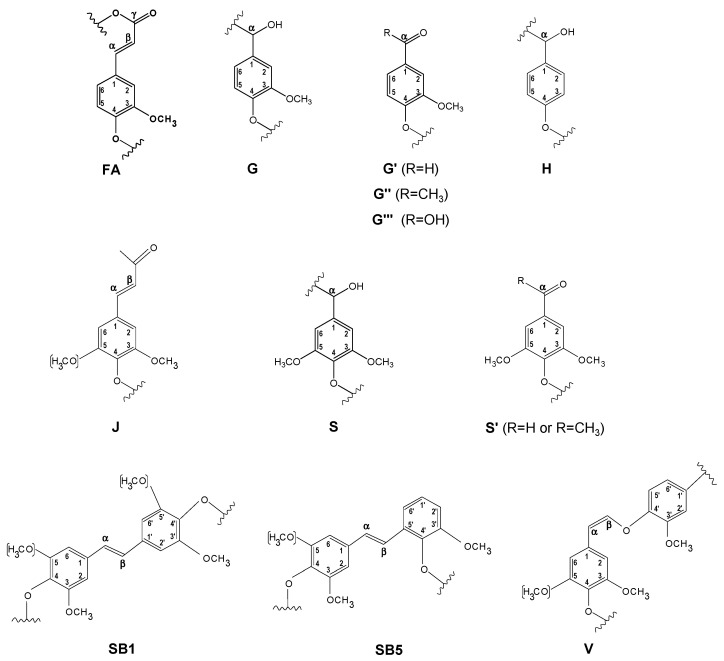
Main lignin substructures identified in aromatic region of orange and olive soda/AQ lignins: (**FA**), ferulate; (**G**), guaiacyl unit; (**G**’), vanillin; (**G’’**), acetovanillona; (**G’’’**), vanillic acid; (**H**), p-hydroxyphenyl unit; (**J**), cinnamyl aldehyde end-groups; (**S**), syringyl unit; (**S’**), syringaldehyde or acetosyringone; (**SB1**), stilbene-β-1’; (**SB5**), stilbene-β-5’; (**V**), vinyl ether.

The ^13^C–^1^H two dimensional nuclear magnetic resonance (2D NMR) spectra of orange and olive soda/AQ lignins are showed in Figure 3 and Figure 4, respectively, including the whole spectra (δ_C_/δ_H_ 0.0–150.0/0.0–9.0) and the spectra corresponding to the oxygenated aliphatic (δ_C_/δ_H_ 45.0–95.0/2.5–6.0 ppm) and the aromatic (δ_C_/δ_H_ 90.0–150.0/5.0–9.0 ppm) regions. The main ^13^C–^1^H lignin correlation signals identified in HSQC spectra are listed in Table 1, assigned according to those reported by different studies [19,20,22,23,24,29,34,35,36,37]. The lignin substructures and carbohydrates identified are represented in Figure 5 and Figure 6.

The non-oxygenated aliphatic region (around δ_C_/δ_H_ 0.0–50.0/0.0–5.0 ppm) exhibited a variety of saturated aliphatic moieties with quite high intensities, especially in soda/AQ-olive lignin spectrum (Figure 4a). Some of these signals could be associated to extractives [38], whereas others could be assigned to groups neighbouring alkene and oxygen-containing groups such as ethers, carbonyl and alcohol, which could originate from lignin degradation [39].

The oxygenated aliphatic region of both spectra displayed the information about the different inter-unit linkages of lignin samples, including those from native and soda/AQ lignin derived linkages (Figure 3b and Figure 4b). The predominant signals corresponding to native lignin linkages observed in both spectra were assigned to β-β’ resinol substructures, including correlations of C_α_–H_α_ (B_α_), C_β_–H_β_ (B_β_) and the double C_γ_–H_γ_ (B_γ_). The resinol substructures with C–C bonds are usually stable to alkaline pulping processes [24,40]. Other signals from native lignin linkages were also visible, although in a lesser extent probably due to its degradation during alkaline pulping. Then, C_β_–H_β_ correlation (C_β_) from β-5’ phenylcoumaran substructures was observed in soda/AQ-orange lignin spectrum (Figure 3b), whereas C_γ_–H_γ_ correlation (B_γ_) was found in both orange and olive soda/AQ lignins spectra (Figure 3b and Figure 4b, respectively). Signals for spirodienones were clearly observed in soda/AQ-orange lignin (Figure 3b), containing C_α_–H_α_ (E_α_) and C_α__’_–H_α__’_ (E_α__’_) correlations, and in a lesser extent in soda/AQ-olive lignin (Figure 4b), whereas C_γ_–H_γ_ correlation signal for cinnamyl alcohol end-groups (I_γ_) was detected in both lignin samples. Regarding β-*O*-4’ substructures, a weak intensity signal of C_α_–H_α_ for β-*O*-4’ substructures (A_α_) was noticed in both spectra, involving S units in soda/AQ-orange and soda/AQ-olive lignins (Figure 3b and Figure 4b, respectively) and also G units in soda/AQ-olive lignin (Figure 4b). C_γ_–H_γ_ (A_γ_) correlations from β-*O*-4’ substructures were also observed in both lignin samples, which in part are overlapped with other signals. This scarce presence of signals attributed to β-*O*-4’ substructures is explained by the preferential β-*O*-4’ linkage breakdown under alkaline conditions [23,24,28], which supports the high phenolic content previously described by ^13^C NMR for both lignins (Section 2.3).

Signals from soda/AQ derived lignin linkages were also observed in the oxygenated aliphatic region of both spectra. Aryl-glycerol substructure (AG), with correlations of C_α_–H_α_ (AG_α_), C_β_–H_β_ (AG_β_) and C_γ_–H_γ_ (AG_γ_), could be tentatively identified in both spectra (Figure 3b and Figure 4b), especially in soda/AQ-orange lignin. This substructure is produced from the non-phenolic β-aryl ether linkage under alkaline pulping processes, especially in soda pulping rather than kraft pulping [24]. C_α_–H_α_ correlation signal of lignin terminal structures with a carboxyl group in C_β_ (Ar–CHOH–COOH; F_α_), which overlaps with C_α_–H_α_ correlation signal of aryl-glycerol substructure, could also be assigned in both spectra. This kind of lignin terminal structures has recently been described during alkaline processes such as kraft pulping of poplar, elm and spruce [19,24,29]. Finally, signals from epiresinol (B’), a diastereomer resulting from the conversion of native resinol (β-β’) substructure during kraft process [35], were also found in both spectra (Figure 3b and Figure 4b). C_α_–H_α_ (B’_α_), C_β_–H_β_ (B’_β_) and C_γ_–H_γ_ (B’_γ_) correlation signals were detected in the case of soda/AQ-orange lignin (Figure 3b), whereas C_γ_–H_γ_ (B’_γ_) in the case of soda/AQ-olive lignin (Figure 4b).

Carbohydrates signals were also observed in the oxygenated aliphatic region of orange and olive soda/AQ-lignin spectra (Figure 3b and Figure 4b, respectively). These signals comprised mainly correlations of xylan chain for C_2_–H_2_ (X_2_), C_3_–H_3_ (X_3_), C_4_–H_4_ (X_4_), and C_5_–H_5_ (X_5_). Moreover, both spectra showed the C-1 cross peak for (1-4) β-d-Xylp of xylan (Figure 3a and Figure 4a).

In the aromatic region of both spectra (Figure 3c and Figure 4c), the characteristic correlation signals of S, G, and H lignin units were seen, in the same way that other hardwoods such as eucalypt, poplar, elm and black locust [19,20,29,40]. The S lignin units presented correlation signals of C_2,6_–H_2,6_ (S_2,6_). C_2,6_–H_2,6_ in oxidized S units (S’_2,6_) with a ketone group (acetosyringone) or aldehyde end-group (syringaldehyde) in C_α_ was also observed. The G lignin units showed correlation signals for C_2_–H_2_ (G_2_), C_5_–H_5_ (G_5_), and C_6_–H_6_ (G_6_). Signals from oxidized G units were also visible, including correlations in soda/AQ-orange lignin attributed to C_2_–H_2_ (G’_2_) and C_6_–H_6_ (G’_6_) in C_α_ oxidized G units bearing an aldehyde-end group (vanillin), C_2_–H_2_ (G’’_2_) and C_6_–H_6_ (G’’_6_) in C_α_ oxidized G units bearing a ketone group (acetovanillone) and C_2_–H_2_ (G’’’_2_) and C_6_–H_6_ (G’’’_6_) in C_α_ oxidized G units bearing a carboxylic group (vanillic acid) (Figure 3c). Some of these oxidized G signals were also observed in soda/AQ-olive lignin spectrum (Figure 4c), confirming the lignin oxidation observed by FTIR (Section 2.2) and ^13^C NMR (Section 3.3) produced during soda/AQ pulping process. In this sense, Prinsen et al. [23] reported a higher lignin oxidation, with an increment between 200−400% of carboxylic groups, during soda/AQ pulping of eucalypt. Lastly, the H lignin units showed correlation signals of C_2,6_–H_2,6_ (H_2,6_) and C_3,5_–H_3,5_ (H_3,5_).

Other native lignin units could also be identified in the aromatic region of both lignin spectra. Then, low intensity signals corresponding to ferulates, including correlations for C_2_–H_2_ (FA_2_), C_α_–H_α_ (FA_α_) and C_β_–H_β_ (FA_β_), were observed in soda/AQ-orange lignin spectrum (Figure 3c). Ferulates can be found in the structure of non-woody plants lignins such as wheat straw and elephant grass [41,42], acylating cell wall carbohydrates and contributing to lignin-carbohydrates cross-coupling reactions, becoming integrally bound into the lignin molecule [43]. Nevertheless, ferulates have been also reported in *Quercus suber* L. [44], and more recently in *Ulmus minor* Mill. [29]. On the other hand, soda/AQ-olive lignin spectrum showed weak correlation signals corresponding to C_β_–H_β_ (J_β_) and C_2,6_–H_2,6_ (J_2,6_) of cinnamaldehyde end-groups (Figure 4c).

Signals from soda/AQ derived lignin linkages were also observed in the aromatic region of both lignin spectra. Correlation signals attributed to C_α_–H_α_ in β1 stilbene (SB1_α_), and C_α_–H_α_ and C_β_–H_β_ in β5 stilbene (SB5_α_ and SB5_β_, respectively) were detected in soda/AQ-orange lignin (Figure 3c), some of them also observed in soda/AQ-olive lignin (Figure 4c). These lignin derived structures have been reported as a degradation product from spirodienone, in the case of β1 stilbene, and β-5’ phenylcoumaran, in the case of β5 stilbene, both via reversed aldol addition during kraft and soda pulping processes of eucalypt and spruce [34,35]. On the other hand, correlation signals associated to C_α_–H_α_ in vinyl-ether were also observed in soda/AQ-orange lignin spectrum (Figure 3c), including correlations from their corresponding two isomers (V_trans_ and V_cis_). Vinyl-ether is normally formed from free phenolic β-*O*-4’ substructures under kraft and soda processes via reversed aldol addition, being described in solubilized lignins during kraft and soda pulping from eucalypt, elm and spruce [24,29].

The quantification of lignin substructures and end-groups (per 100 aromatic units), aromatic units (molar percentage) and S/G ratios of soda/AQ lignins are presented in Table 2. As previously mentioned, the β-*O*-4’ alkyl-aryl ether linkages cleavage is the main delignification reaction produced in lignin under alkaline pulping conditions, resulting in solubilized lignin with high phenolic content and low molecular weight [23,24,28]. C–C bonds from phenylcoumaran (β-5’) can also be degraded under these alkaline conditions, whereas from resinol (β-β’) are usually more stable [23,24]. In this line, both lignins showed a higher content of β-β’ resinol substructures (4.3 and 3.5 linkages per 100 aromatic units for orange and olive soda/AQ lignins, respectively), compared to the other linkages, i.e., β-*O*-4’ alkyl-aryl ether (0.8 linkages per 100 aromatic units for soda/AQ-orange lignin; and 1.3 linkages per 100 aromatic units for soda/AQ-olive lignin) and β-5’phenylcoumaran (0.3 and 0.2 linkages per 100 aromatic units for orange and olive soda/AQ lignins, respectively). In this sense, Prinsen et al. [23] also described an enrichment of β-β’ resinol substructures together with a scarce or null abundance of both β-*O*-4’ and β-5’ phenylcoumaran substructures when eucalypt was subjected to soda/AQ pulping. Similar tendency has also been reported by Zhao et al. [24] during soda pulping of spruce and by Domínguez-Robles et al. [22] during soda pulping of agricultural residues such as wheat straw and barley straw as well as soda/AQ pulping of fast growing plants such as *L. leucocephala* and *C. proliferus*. Spirodienone (0.9 and 0.6 linkages per 100 aromatic units for soda/AQ-orange and soda/AQ-olive lignins, respectively), aryl-glycerol (2.1 linkages per 100 aromatic units for soda/AQ-orange lignin and 0.5 linkages per 100 aromatic units for soda/AQ-olive lignin) and Ar–CHOH–COOH (3.1 and 0.5 linkages per 100 aromatic units for orange and olive soda/AQ lignins, respectively) substructures were quantified. Nevertheless, the quantities determined for Ar–CHOH–COOH and spirodienones substructures could be overvalued by the overlap of their signals with those belonging to aryl-glycerol and epiresinols, respectively. Cinnamyl alcohol end-groups were also quantified (1.1 and 0.9 linkages per 100 aromatic units for orange and olive soda/AQ lignins, respectively).

The S/G ratio calculated for both soda/AQ lignins showed a high content of S lignin units (4.6 and 3.1 for orange and olive soda/AQ lignins, respectively). It is well known the better solubilization of S lignin units during alkaline pulping processes such as kraft and soda/AQ processes, phenomenon already described for eucalypt lignin solubilized during these pulping technologies [23] and different gramineae and fabaceae soda lignins [22]. Abundance of stilbene substructures were also calculated in both lignins, displaying higher quantities in soda/AQ-olive lignin (2.8 and 3.0 linkages per 100 aromatic units for stilbene-β1 and stilbene-β5, respectively) compared to soda/AQ-orange lignin (0.9 linkages per 100 aromatic units for stilbene-β1 and 1.5 linkages per 100 aromatic units for stilbene-β5). Finally, ferulates and vinyl-ether were quantified in soda/AQ-orange lignin (0.5 and 1.0 linkages per 100 aromatic units, respectively), whereas cinnamaldehyde end-groups were measured in soda/AQ-olive lignin (0.8 linkages per 100 aromatic units).

### 2.5. SEC Analysis

**Figure 7:**
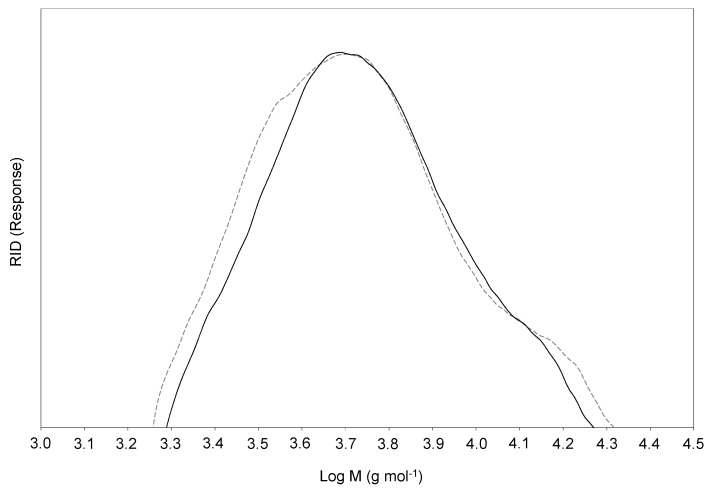
Molecular weight distribution of soda/AQ-orange lignin (discontinuous line) and soda/AQ-olive lignin (continuous line).

The molecular weight distributions of orange and olive soda/AQ lignins are shown in Figure 7. Weight-average (*Mw*) and number-average (*Mn*) molecular weights, and polydispersity (*Mw*/*Mn*) values were calculated from them (Table 3). The low molecular weight values observed for both lignins (6.4 KDa and 6.2 KDa for soda/AQ-orange and soda/AQ-olive lignins, respectively) clearly showed the high degradation and depolymerization of the lignin polymer during the soda/AQ pulping process. This fact is in accordance with the major cleavage of β-*O*-4’ alkyl-aryl ether linkages observed by 2D NMR (Section 2.4). Prinsen et al. [23] described a strong decrease in the molecular weight values of solubilized lignins from eucalypt due to a broad breakdown of aryl ether linkages produced during kraft and soda/AQ pulping processes. In the same way, Tejado et al. [28] reported low molecular weight values together with an extended cleavage of β-*O*-4’ aryl ether linkages in solubilized lignins from flax during soda/AQ process. Moreover, the solubilized lignin fragments with small molecular weights resulting from degradation of the lignin macromolecule during the alkaline soda/AQ were also more uniformly distributed, as polydispersity values reflected (Table 3).

### 2.6. Thermal Analysis

**Figure 8:**
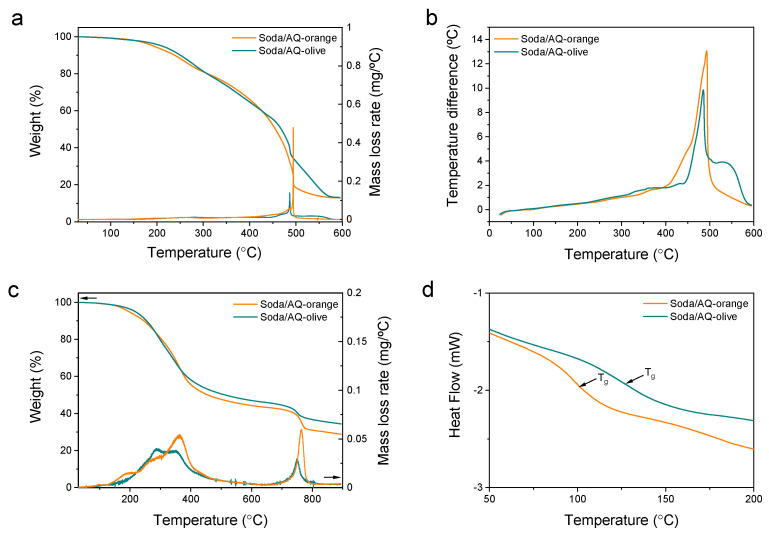
Thermogravimetry (TG) curves in air (**a**) and in nitrogen (**c**) and differential thermal analysis (DTA) curves in air (**b**) of orange and olive soda/AQ lignins. Differential scanning analysis (DSC) curves in nitrogen (**d**). The glass transitions (T_g_) are indicated by arrows.

Both soda/AQ lignins were subjected to thermal analysis in air and under nitrogen for further structural and compositional characterization. Figure 8 shows that the orange and olive soda/AQ lignins display very similar thermal properties with only small differences. For instance, the thermogravimetric (TG) curves in air reveal that the onset temperature (T_on_) of thermal degradation is lower for orange (160 °C) than for olive (215 °C) lignin (Figure 8a). Following a gradual thermal degradation step, pronounced weight loss takes place with a maximum rate at 487 °C (soda/AQ-olive lignin) and 495 °C (soda/AQ-orange lignin). This step is generally attributed to the oxidative combustion of char formed during the previous thermal degradation process [45]. The oxidative combustion is also corroborated by differential thermal analysis (DTA) that shows an exothermic peak in the same temperature range (~490 °C) (Figure 8b). The residual weight at 600 °C is 11% for both lignins, which suggests the presence of inorganic compounds. These inorganic compounds are present in the raw materials as well as those used in soda/AQ pulping (e.g., NaOH) but also to the high amount of Na_2_SO_4_ salts formed during the acid precipitation step. Interestingly, soda/AQ-olive lignin displays a further high temperature combustive process at 530 °C, as suggested by the shoulder in Figure 8b.

Under nitrogen, the TG curves show two distinct weight loss steps in the region of 200–400 °C and around 760 °C. The latter can be possibly ascribed to the inorganic fraction of the lignins as suggested by an endothermic peak in the corresponding DTA curves (Figure 8c). Like in air, T_on_ of the thermal degradation of soda/AQ-olive lignin is higher than that of soda/AQ-orange lignin (230 vs. 162 °C) as well as of different gramineae and fabaceae soda lignins [22]. Furthermore, differential thermogravimetry (DTG) reveals the maximum weight loss rate at 362 °C for soda/AQ-orange lignin, whereas soda/AQ-olive lignin has the maximum rate at 290 and 350 °C (Figure 8c). This DTG temperature profile may well be explained by the chemical composition of these two lignins. Soda/AQ-orange sample has a higher lignin (75.1%) and a lower carbohydrate (6.4%) content than soda/AQ-olive sample (69.9 and 16.3%, respectively). Due to their cross-linked and branched aromatic structure, lignins usually have higher thermal stability than carbohydrates, especially hemicelluloses [22,45]. Moreover, the higher amount of more thermostable β-β’ versus weaker β-O-4’ linkages in soda/AQ-orange lignin may also contribute to the observed higher stability of this lignin [46].

The differential scanning calorimetry (DSC) curves show the glass transition temperature (T_g_) of soda/AQ-orange lignin at 100 °C, while the T_g_ of soda/AQ-olive lignin is slightly higher at 126 °C (Figure 8d). While in general it is well-known that T_g_ correlates with the molecular weight of polymers and lignins [47], in the present case it may not explain the observed difference in the glass transition temperature given that the *Mw* values of both lignins are fairly similar (6.2 and 6.4 kDa). In addition, other molecular parameters such as branching, cross-linking, molecular flexibility, *Mw*/*Mn* and carbohydrate contamination also influence the T_g_ of a given lignin. The T_g_ values reported here are lower than those reported for other lignins, such as from soda/AQ flax (138 °C) [28], kraft elm (137 °C) [29] or organosolv black locust (155 °C) [19].

Overall, the orange and olive soda/AQ lignins show relatively higher thermal stability both under nitrogen and in air as compared to lignins extracted from other woody biomass by alkaline processes such as poplar and elm lignins solubilized during kraft pulping [20,29] and lignins during soda pulping of wheat straw and barley straw as well as during soda/AQ pulping of *L. leucocephala* and *C. proliferus* [22].

### 2.7. Antioxidant Properties

The structure, functional groups, molecular weights, etc., of lignins may influence their antioxidant activity (i.e., their ability to act as radical scavengers) promoting their use as natural additives to replace synthetic compounds in pharmaceuticals, cosmetics, food and polymeric formulations [48]. The relationship between lignin structure and antioxidant activity has been extensively evaluated [49,50]. Overall, it has been reported that having higher phenolic hydroxyl content, low molecular weight and narrow distribution seem to be beneficial for antioxidant activity of lignin [48]. Taking into account these considerations, the high content of phenolic units observed for both soda/AQ lignins by ^13^C NMR (Section 2.3) as well as their low molecular weight and polydispersity values (Section 2.5) could lead to good antioxidant activities. The ABTS^•+^ radical scavenging activity was quantified for both soda/AQ lignins (Table 4), showing antioxidant activities of 149.7 and 202.4 mg TE g^−1^ for soda/AQ-orange lignin and soda/AQ-olive lignin, respectively. The higher antioxidant capacity of soda/AQ-olive lignin could be due to its slightly lower molar weight and narrow polydispersity, in spite of containing slightly lower amount of phenols content. Nevertheless, there are other structural factors that may provide antagonist effects on the antioxidant capacity of lignin. Thus, the abundance of ortho-methoxyl groups (guaiacyl and syringyl) and α-methylene groups (α-CH2) improved antioxidant capacity, while carbonyl groups and double C=C bonds in the α-position showed a negative effect according to some authors [51]. Similar antioxidant capacity has been reported for other lignins. Thus, Ratanasumarn and Chitpraser [52] reported Trolox equivalent antioxidant capacity of 221–273 mg TE g^−1^ for different lignin extracts from alkaline-treated sugarcane. In the same way, Qazi et al. [53] described a significant variation in the antioxidant activity (35–277 mg TE g^−1^) when the ABTS^•+^ radical scavenging activity was evaluated on several pyrolytic lignins. However, Juttuporn et al. [54] reported lower antioxidant capacity (35–83 mg TE g^−1^) for sugarcane bagasse lignin extracts obtained by steam explosion and ultrasound-assisted extraction or by different solvent extractions. On the other hand, kraft lignin, more similar to soda/AQ lignin than the previously cited, have been reported to have an antioxidant activity of 30–68% (percentage of inhibition, measured with 2,2-diphenyl-1-picrylhydrazyl (DPPH) instead of ABTS^•+^) [51,55,56], which can be comparable with thus showed by soda/AQ lignin in our work (35–47%, for 12.5mg L^−1^ of lignin). Finally, García et al. [57] reported antioxidant capacities between 16.1% and 46.8% (percentage with respect to the inhibition observed for the DPPH with Trolox after 60 min of reaction) for lignin with molar weight (*Mw*) between 1.1 KDa and 5.6 KDa, extracted by different methods from *Miscanthus sinensis*. They observed a clear relation between antioxidant capacity and the molar weight and polydispersity, apart from phenolic content. Moreover, among these lignins, they study a soda lignin with similar molar weight than those presented in our work (*Mw* 5.7 KDa) which showed an antioxidant capacity of 16.1%, similar to those observed for soda/AQ-orange and -olive lignins: 15.3% and 20.5% (percentage with respect to the inhibition observed for the ABTS^•+^ with Trolox after 6 min of reaction).

This antioxidant property could be exploited in mixtures with other synthetic polymers (e.g., polyethylene, polypropylene, poly (vinyl alcohol)) with the objective to reduce their oxidative degradation [58,59,60], particularly those governed by radical mechanisms. Then, commercial antioxidants such as hindered phenols and amines traditionally applied to polymers could be partially substituted by these natural soda/AQ lignins. Furthermore, lignin could be used also in pharmaceutical, cosmetic, food and packaging industries as a substitute of commonly used cytotoxic synthetic antioxidant like butylated hidroxytoluene (BHT) or butylated hydroxyanisole (BHA) since it has shown higher antioxidant power according to different authors [51,55,56,61,62].

Together with the antioxidant activity, the relatively high thermal stability described for both soda/AQ lignins (Section 2.6) make them potential additives to improve the thermo-oxidation stability of synthetic polymers [63]. This could particularity improve the flame resistance performance of synthetic polymers [64], wherein these soda/AQ lignins might become potential substitutes of organic flame retardants like halogenated bisphenol A or polybrominated diphenyl ethers [65,66]. Nevertheless, at this stage only conjectures can be made about the applicability of these orange and olive soda/AQ lignins as environmentally benign, flame retardant antioxidants, while these properties need to be verified experimentally in the mentioned polymer mixtures.

## 3. Materials and Methods

### 3.1. Raw Materials and Chemicals

Orange tree (*Citrus sinensis*) and olive tree (*Olea europea*) pruning residues were kindly provided by Universidad de Córdoba and Universidad de Jaén, respectively. The samples were chipped, homogenized, and stored until their use. On average, the orange tree pruning residue presented the following composition: 3.6%, extractives; 3.4%, ash; 19.9%, lignin; and 73.2% holocellulose [1]; whereas the composition for olive tree pruning residue was: 8.0% extractives; 1.4%, ash; 20.7% lignin; 89.2% and holocellulose [6].

All chemicals were reagent-grade and were purchased from Merck (Barcelona, Spain), Panreac (Barcelona, Spain) or Sigma-Aldrich (Madrid, Spain).

### 3.2. Pulps and Lignins Production

Pulp from orange tree pruning residue was obtained according to Fillat et al. [1]. The material was cooked with soda/anthraquinone (soda/AQ) under the following conditions: 185 °C, 60 min, 20% (*w*/*w*) NaOH, 1% (*w*/*w*) AQ (both oven-dried material) and 8:1 liquid/solid ratio. Regarding olive tree pruning residue the cooking conditions employed for pulp production were: 175 °C, 120 min, 15% (*w*/*w*) NaOH, 1% (*w*/*w*) AQ (both oven-dried material) and 8:1 liquid/solid ratio [6]. Resulting soda/AQ pulps were filtered, collecting the black liquors for lignin precipitation. Orange soda/AQ pulp showed a kappa number, viscosity, and brightness values of 22.0, 430 mL g^−1^ and 33.5% ISO, respectively, whereas olive soda/AQ pulp showed a kappa number and viscosity values of 38.7 and 794 mL g^−1^ and a brightness of 18.4% ISO.

Orange and olive soda/AQ lignins (referenced as soda/AQ-orange and soda/AQ-olive, respectively) were extracted from recovered black liquors by acid precipitation. Then, the pH of the black liquors was lowered to 2.5 by slow addition of sulphuric acid (98% *w*/*w*) and left under stirring for 30 min. The precipitated lignins were filtered and washed twice with acidified water (pH 2.5) and dried at room temperature.

### 3.3. Lignins Characterization

Chemical composition of soda/AQ lignins was examined according to the Laboratory Analytical Procedures for biomass analysis from the National Renewable Energies Laboratory [67], employing the protocol NREL/TP-510-42618. After the acid hydrolysis of lignin samples, the acid insoluble solid residue (klason lignin) was recovered, whereas the liquid fraction was examined for carbohydrates content by high-performance liquid chromatography (1260 HPLC, Agilent, Waldbronn, Germany, equipped with a G1362A refractive index (RI) detector and an Agilent Hi-PlexPb column) [30]. Mean values and standard deviations were calculated from the triplicates.

The total phenols content of soda/AQ lignins was evaluated according to a slightly modified version of the Folin-Ciocalteau protocol [30]. Then, the absorbance of a mixture with Folin-Ciocalteau solution, Na_2_CO_3_ and lignin samples (previously dissolved in dimethylsulfoxide) was measured at 760 nm in a UV-Vis spectrophotometer (Lambda 365, PerkinElmer, Boston, MA, USA). The total phenols content was calculated from a calibration curve prepared from a standard solution of gallic acid (1–20 mg L^−1^) and expressed as g gallic acid equivalent (GAE) mg^−1^ of lignin (on a dry basis). Mean values and standard deviations were calculated from the triplicates.

FTIR spectra of soda/AQ lignins were acquired by a JASCO FT/IR 460 Plus spectrometer (Jasco, Japan), with an accessory single reflection diamond, working at a resolution of 1 cm^−1^, 100 scans, and a spectral range of 4000–600 cm^–1^ [30].

Solid-state ^13^C nuclear magnetic resonance (^13^C NMR) analyses of soda/AQ lignins were carried out in a Bruker Avance III 400MHz (Bruker, Billerica, MA, USA) at 100.64 MHz with the cross polarization/magic angle spinning (CP/MAS) technique at the conditions described by Jiménez-López et al. [30].

^13^C–^1^H two dimensional nuclear magnetic resonance (2D NMR) analyses of soda/AQ lignins were recorded in a Bruker AVANCE 500 MHz (Bruker, Billerica, MA, USA) with a z-gradient double resonance probe. Soda/AQ lignins were dissolved in deuterated dimethylsulfoxide (DMSO-*d*_6_) and HSQC (heteronuclear single quantum correlation) experiments were recorded at the conditions reported by Eugenio et al. [29] and Martín-Sampedro et al. [19]. Residual DMSO (at δ_C_/δ_H_ 39.6/2.5 ppm) was used as an internal reference.

Size exclusion chromatography (SEC) of soda/AQ lignins was conducted on a HPLC (1260 HPLC, Agilent, Waldbronn, Germany, equipped with a G1362A refractive index (RI) detector and two columns PLgel 10µm MIXED B 300 × 7.5 mm). *N*,*N*-dimethylformamide (DMF) was pumped as mobile phase at the conditions described by Jiménez-López et al. [30]. Columns were calibrated with polystyrene standards (peak of average molecular weights of 570, 8900, 62,500, 554,000, Sigma-Aldrich, San Luis, MO, USA).

### 3.4. Thermal Lignins Characterization

Thermogravimetric analysis (TGA) and differential thermal analysis (DTA) of soda/AQ lignins were carried out in air and nitrogen (SDT Q600, TA Instruments, New Castle, DE, USA), using a heating rate of 10 °C min^−1^ [20]. In addition, differential scanning calorimetry (DSC) was carried out under nitrogen (Q2000 calorimeter, TA Instruments), using a heating rate of 20 °C min^−1^ [19]. In order to remove any previous thermal history from soda/AQ lignins, the samples were dried (60 °C for 48 h) and a heating and cooling cycle from 20 to 160 °C in the DSC instrument was performed [68].

### 3.5. Antioxidant Activity of Lignins

The antioxidant activity of soda/AQ lignins was performed using Trolox equivalent antioxidant capacity methods according to Re et al. [69], with slight modifications [52]. ABTS^+•^ was produced by the reaction between a stock solution of 2,2′-azino-bis(3-ethylbenzthiazoline-6-sulphonic acid) diammonium salt (ABTS) and potassium persulfate. Prior to use, the ABTS^+•^ stock solution was diluted with phosphate buffer saline to get an absorbance of 0.7 ± 0.02 at 734 nm. Then, 1 mL of the ABTS^+•^ stock solution was mixed with 10 μL sample (1 mg mL^−1^) or control (buffer). The absorbance of the reaction mixture was measured at 734 nm during 6 min in a UV–Vis spectrophotometer Lambda 365 (PerkinElmer, Boston, MA, USA). Trolox was employed as a standard and results were showed in mg of Trolox equivalent (TE) g^−1^ lignin. Mean values and standard deviations were calculated from the triplicates.

## 4. Conclusions

In order to increase the competitive and sustainable production of energy and high added-value products from carbohydrates contained in lignocellulosic materials, including agroforestry residues, the valorization of lignin-enriched residues generated during these transformation processes is crucial. In addition, the use of these lignin-enriched residues will also contribute for the establishment of the circular bioeconomy, which seeks to maximize the usage and value of all raw materials, products and residues. Nevertheless, depth knowledge of lignin is necessary to define its valorization route. In this study, residual lignins solubilized during soda/AQ pulping of orange and olive tree pruning residues, two of the most abundant agroforestry residues generated in Spain, were isolated and chemical, thermal and antioxidant properties analyzed. Both lignins showed extensive β-*O*-4’ linkages degradation, as revealed ^13^C–^1^H two dimensional nuclear magnetic resonance analyses. Consequently, a high OH phenolic content, inferred by solid state ^13^C NMR, and low molecular weight and polydispersity values, showed by size exclusion chromatography, were observed for both lignins. In addition, lignins displayed a high proportion of syringyl units, containing different native as well as soda/AQ lignin derived units. Based on these chemical properties, orange and olive lignins exhibited relatively high thermal stability and good antioxidant properties. This antioxidant capacity could be used in blends with other synthetic polymers in order to reduce their oxidative degradation. Moreover, the relatively high thermal stability described for both lignins makes them potential additives to improve the thermo-oxidation stability of synthetic polymers.

## Figures and Tables

**Table 1:** Assignment of main lignin and carbohydrates ^13^C-^1^H correlation peaks in the HSQC spectra of soda/AQ lignins.

δ_C_/δ_H_ (ppm)	Assignment
49.7/3.38	C_β_–H_β_, epiresinol substructures (**B’**)
53.3/3.49	C_β_–H_β_, phenylcoumaran substructures (**C**)
54.2/3.08	C_β_–H_β_, resinol substructures (**B**)
56.1/3.72	C–H, methoxyls (**MeO**)
60.6/3.38–3.64	C_γ_–H_γ_, β-*O*-4’ substructures (**A**)
61.8/4.11	C_γ_–H_γ_, cinnamyl alcohol end groups (**I**)
62.7/3.65	C_γ_–H_γ_, phenylcoumaran substructures (**C**)
63.3/3.23–3.90	C_5_–H_5_, xylan
63.3/3.19–3.28	C_γ_–H_γ_, aryl-glycerol (**AG**)
69.5/3.11–3.57	C_γ_–H_γ_, epiresinol substructures (**B’**)
70.5/3.73–4.09	C_γ_–H_γ_, epiresinol substructures (**B’**)
71.3/3.76–4.18	C_γ_–H_γ_, resinol substructures (**B**)
71.6/4.79	C_α_–H_α_, β-*O*-4’ **G** unit (**A**)
72.2/4.88	C_α_–H_α_, β-*O*-4’ **S** unit (**A**)
72.8/3.08	C_2_–H_2_, xylan
74.0/4.45	C_α_–H_α_, aryl-glycerol (**AG**)
74.1/3.28	C_3_–H_3_, xylan
74.3/4.37	C_α_–H_α_, Ar–CHOH–COOH units (**F**)
75.6/3.49	C_β_–H_β_, aryl-glycerol (**AG**)
75.9/3.55	C_4_–H_4_, xylan
81.7/4.74	C_α_–H_α_, spirodienone substructures (**E**)
81.8/4.76	C_α_–H_α_, epiresinol substructures (**B’**)
85.3/4.74	C_α__’_ –H_α__’_, spirodienone substructures (**E**)
85.5/4.63	C_α_–H_α_, resinol substructures (**B**)
87.4/4.31	C_α_–H_α_, epiresinol substructures (**B’**)
101.6/4.30	C-1, (1-4) β-D-Xylp
104.3/6.69	C_2,6_–H_2,6_, S units (**S**)
106.2/7.12	C_2,6_–H_2,6_, in cinamaldehyde end-groups (**J**)
106.7/7.32	C_2,6_–H_2,6_, oxidized (H–C_α_=O or H_3_C–C_α_=O) S units (**S’**)
109.8/5.54	C_α_–H_α_, isomer cis of vinil ether (**V**)
111.0/6.88	C_2_–H_2_, G units (**G**)
111.0/7.38	C_2_–H_2_, oxidized (H–C_α_=O) G units (**G’**)
111.7/7.31	C_2_–H_2_, ferulate (**FA**)
112.3/7.51	C_2_–H_2_, oxidized (H_3_C–C_α_=O) G units (**G’’**)
112.4/6.13	C_α_–H_α_, isomer trans of vinyl ether (**V**)
113.2/7.44	C_2_–H_2_, oxidized (HO–C_α_=O) G units (**G’’’**)
114.3/6.48	C_β_–H_β_, ferulate (**FA**)
115.0/6.74	C_3,5_–H_3,5_, p-hydroxyphenyl (**H**)
115.2/6.42–6.81	C_5_–H_5_, G units (**G**)
119.6/6.78	C_6_–H_6_, G units (**G**)
120.6/7.21	C_β_–H_β_, stilbene (**SB5_β_**)
123.7/7.52	C_6_–H_6_, oxidized (H_3_C–C_α_=O) G units (**G’’**)
123.8/7.49	C_6_–H_6_, oxidized (HO–C_α_=O) G units (**G’’’**)
126.2/6.97	C_α_–H_α_, stilbene (**SB1_α_**)
126.5/7.43	C_6_–H_6_, oxidized (H–C_α_=O) G units (**G’**)
126.9/6.80	C_β_–H_β_, cinnamaldehyde end-groups (**J**)
128.4/7.17	C_2,6_–H_2,6_, p-hydroxyphenyl (**H**)
128.8/7.13	C_α_–H_α_, stilbene (**SB5_α_**)
144.7/7.46	C_α_–H_α_, ferulate (**FA**)

**Table 2:** Abundance of lignin substructures and end-groups (per 100 aromatic units) and aromatic units (molar percentage) from integration of ^13^C-^1^H correlation peaks in the HSQC spectra of soda/AQ lignins.

	Soda/AQ-Orange	Soda/AQ-Olive
β-*O*-4’ (**A**)	0.8	1.3
Resinols (**B**)	4.3	3.5
Phenylcoumarans (**C**)	0.3	0.2
Spirodienones (**E**)	0.9	0.6
Arylglicerol (**AG**)	2.1	0.5
Ar−CHOH−COOH (**F**)	3.1	0.5
Ferulates (**FA**)	0.5	-
cinnamyl alcohol end-groups (**I**)	1.1	0.9
cinnamaldehyde end-groups (**J**)	-	0.8
Stilbene (**SB1**)	0.9	2.8
Stilbene (**SB5**)	1.5	3.0
Vinyl-ether (**V**)	1.0	-
H (%)	0.8	0.7
G (%)	17.7	24.1
S (%)	81.5	75.2
S/G ratio	4.6	3.1

Soda/AQ-orange, solubilized orange lignin recovered from soda/AQ black liquor; soda/AQ-olive, solubilized olive lignin recovered from soda/AQ black liquor. Abundance of β-*O*-4’, resinols, phenylcoumarans, spirodienones, arylglicerol, and Ar**−**CHOH**−**COOH substructures was estimated by 2D-NMR from C_α_–H_α_ correlations. Cinnamyl alcohol end-groups using C_γ_–H_γ_ correlations, respectively; Cinnamaldehyde end-groups using C_β_–H_β_ correlations; Ferulates, vinyl-ether and stilbenes (SB1 and SB5) using C_α_–H_α_ correlations; C_2,6_–H_2,6_ correlations from S units; and C_2_–H_2_ correlations from G units were used to estimate the S/G lignin ratios.

**Table 3:** Weight average (*Mw*) and number-average (*Mn*) molecular weights and polidispersity (*Mw*/*Mn*) of soda/AQ lignins. *Mw* and *Mn* are given in Da.

	Soda/AQ-Orange	Soda/AQ-Olive
*Mw*	6430	6280
*Mn*	4890	4980
*Mw/Mn*	1.31	1.26

Soda/AQ-orange, solubilized orange lignin recovered from soda/AQ black liquor; soda/AQ-olive, solubilized olive lignin recovered from soda/AQ black liquor.

**Table 4:** Trolox equivalent antioxidant capacity of soda/AQ lignins.

	Soda/AQ-Orange	Soda/AQ-Olive
mg TE g^−1^ lignin	149.7 ± 1.2	598.2 ± 4.7
mM TE g^−1^ lignin	202.4 ± 5.1	808.9 ± 20.3

Soda/AQ-orange, solubilized orange lignin recovered from soda/AQ black liquor; soda/AQ-olive, solubilized olive lignin recovered from soda/AQ black liquor.

## Data Availability

Not applicable.
